# Biodiversity and ecosystem services require IPBES to take novel approach to scenarios

**DOI:** 10.1007/s11625-016-0354-8

**Published:** 2016-02-09

**Authors:** Marcel T. J. Kok, Kasper Kok, Garry D. Peterson, Rosemary Hill, John Agard, Stephen R. Carpenter

**Affiliations:** 1 PBL Netherlands Environmental Assessment Agency, The Hague, The Netherlands; 2 Soil Geography and Landscape Group, Wageningen University, Wageningen, The Netherlands; 3 Stockholm Resilience Centre, Stockholm University, Stockholm, Sweden; 4 CSIRO Land and Water, Division of Tropical Environments and Societies, James Cook University, Cairns, Australia; 5 Department of Life Sciences, University of the West Indies, Saint Augustine, Trinidad and Tobago; 6 Center for Limnology, University of Wisconsin-Madison, Madison, WI USA

**Keywords:** IPBES, Scenarios, Biodiversity, Ecosystem services, Cross-scale

## Abstract

What does the future hold for the world’s ecosystems and benefits that people obtain from them? While the Intergovernmental Platform on Biodiversity and Ecosystem Services (IPBES) has identified the development of scenarios as a key to helping decision makers identify potential impacts of different policy options, it currently lacks a long-term scenario strategy. IPBES will decide how it will approach scenarios at its plenary meeting on 22–28 February 2016, in Kuala Lumpur. IPBES now needs to decide whether it should create new scenarios that better explore ecosystem services and biodiversity dynamics. For IPBES to capture the social-ecological dynamics of biodiversity and ecosystem services, it is essential to engage with the great diversity of local contexts, while also including the global tele-coupling among local places. We present and compare three alternative scenario strategies that IPBES could use and then suggest a bottom-up, cross-scale scenario strategy to improve the policy relevance of future IPBES assessments. We propose five concrete steps as part of an effective, long term scenario development process for IPBES in cooperation with the scientific community.

## Envisioning the future of biodiversity and ecosystem services

What does the future hold for the world’s ecosystems and benefits that people obtain from them? To help provide an answer to that question, the Intergovernmental Platform on Biodiversity and Ecosystem Services (IPBES) was established in 2012 by the United Nations to become the leading intergovernmental body for assessing the state of the planet’s biodiversity, its ecosystems, and the essential services they provide to society. While IPBES has identified the development of scenarios as a key to helping decision makers identify potential impacts of different policy options, it currently lacks a long term scenario strategy. IPBES will decide how it will approach scenarios at its plenary meeting 22–28 February 2016, in Kuala Lumpur.^1^

Scenarios have been widely used in global environmental assessments. A scenario is a plausible and often simplified description of how the future may develop, based on a coherent and internally consistent set of assumptions about key driving forces and their relationships (Millennium Ecosystem Assessment 2005a). Scenario development has been used in global assessments to focus scientific investigation, integrate different models and data and evaluate policies. However, scenario processes have not often been explicitly designed to achieve these goals, and their success in achieving them has been uneven. Political support for scenario-approaches in other assessments has also been mixed and mandates for scenario development in global assessments contested (Feldman and Biggs 2012).

IPBES needs to decide how to build upon existing material and whether it should create new scenarios that better explore ecosystem services and biodiversity dynamics. For IPBES to capture the social-ecological dynamics of biodiversity and ecosystem services, it is essential to engage with the great diversity of local contexts, while also including the global tele-coupling among local places (Liu et al. 2015). As IPBES aims to include indigenous and local knowledge (ILK) in its assessments, this also requires local engagement. While global environmental scenarios have been developed for diverse topics including energy, agriculture and climate, they share many assumptions, models, and data (Van Vuuren et al. 2012). These scenarios have however been dominated by issues related to climate change and have ecological dynamics not well integrated (Cumming et al. 2005). Consequently, IPBES has to consider what type of effort it should make to produce scenarios that address the cross-scale social-ecological dynamics of biodiversity and ecosystem services. We present and compare three alternative scenario strategies that IPBES could use to overcome these issues, and then suggest a cross-scale scenario strategy.

## What type of scenarios for IPBES?

Global assessments have typically developed scenarios to perform three related functions: focus scientific investigation and synthesis, integrate disparate models and data, and evaluate policies.

IPBES can use scenarios to coordinate and align scientific analysis by defining a diverse but limited set of future trajectories to use as inputs for scientific analyses. Scenarios can define inputs to models, policy analyses or comparisons, ensuring that different analyses are comparable and address a minimum shared set of issues. Such a knowledge base can be used to improve the robustness and relevance of future IPBES assessments. Narrative scenarios can also challenge the modelling community to develop new capabilities (Peterson et al. 2003).

Scenario development is an iterative process that can be used to integrate multiple disparate data sources, knowledge systems and models. Scenarios can integrate quantitative models of climate and ecological dynamics with qualitative analysis of processes that are not modelled or well understood, such as shifts in values, diets, or governance. Scenario development methods that use participatory modelling and mapping can also bring ILK into assessments, which is a priority for IPBES (Robinson et al. 2016).

Scenarios can be used to analyse the consequences of distinct and different choices or policies. Such analysis can assess the strengths and weaknesses of existing and future policies, as well as their robustness to future uncertainty. Furthermore, by connecting multiple domains of knowledge to a greater extent than integrated assessment models, scenarios can expand the diversity of novel policies and strategies that may provide opportunities for policy development or social innovation (Carpenter et al. 2006).

**Fig. 1:**
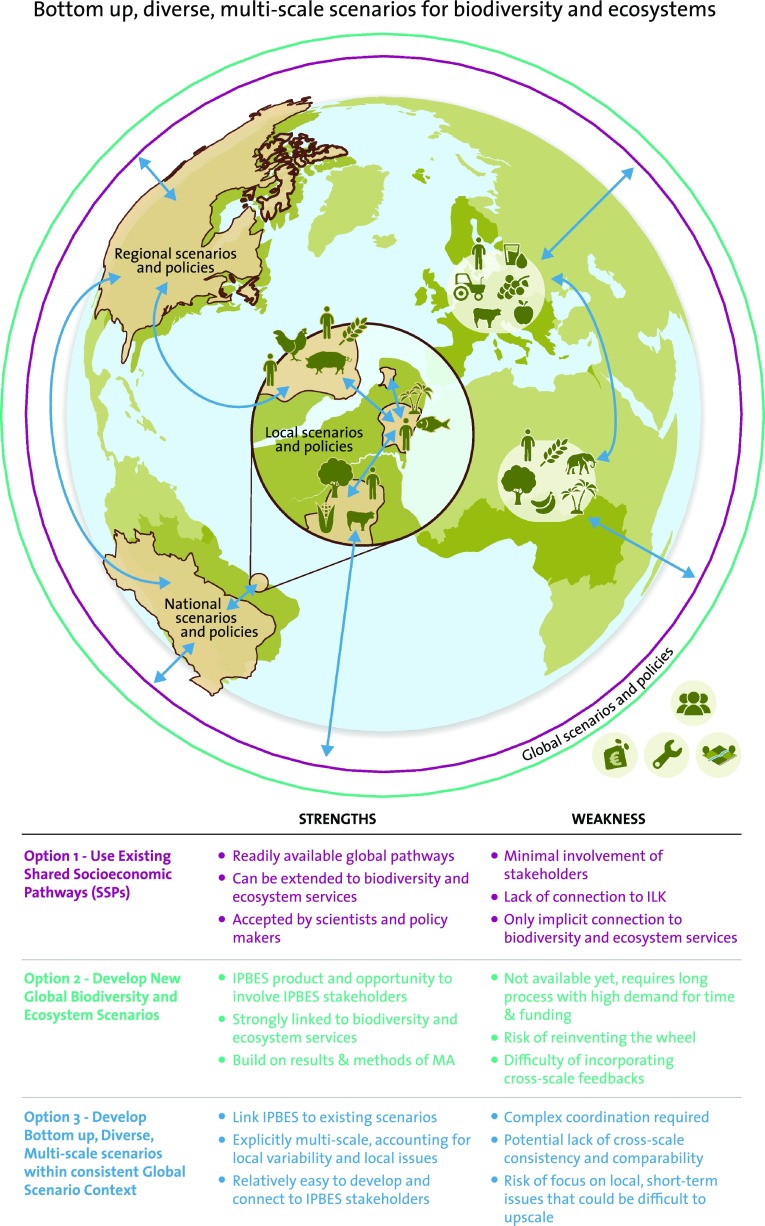
Strengths and weaknesses of the 3 options

What types of scenarios should IPBES develop to best perform these three functions? Based upon previous experience we believe that there are three main options (see also Fig. 1).

### *Option 1:* IPBES uses the most recent set of global scenarios for climate research and extends them for biodiversity and ecosystem services

The recently published global shared socioeconomic pathways (SSPs) (O’Neill et al. 2015) could provide a basis for the preparation of global IPBES scenarios. Developed for the analysis of climate change adaptation and mitigation, the five SSPs consist of stories that span a range of future trajectories of economic, social, institutional, and organisational variables. They have been constructed to enable their extension to non-climate issues, as well as to application at sub-national level (Absar and Preston 2015). However, using these global pathways to project changes in biodiversity and ecosystem services at multiple scales is a substantial undertaking that may oversimplify local social-ecological feedbacks and land-use dynamics that are critical for changes in biodiversity and ecosystem services (Oteros-Rozas et al. 2015; Reid et al. 2006).

### *Option 2:* IPBES develops new global scenarios

IPBES could develop new scenarios focussed on how humanity benefits from and is reshaping biodiversity and ecosystem services. IPBES could build upon the methods developed by and lessons learned from the Millennium Ecosystem Assessment (MA), which was the first global assessment of ecosystem services (Millennium Ecosystem Assessment 2005a, b). The scenarios developed by the MA are less suitable as they are now a decade old and have not been widely used within either policy or scientific contexts. Developing new global scenarios would address the needs of IPBES, but would require substantial investments, including the development of new, multi-scale models and datasets to account for social-ecological tele-connections and cross-scale feedbacks that strongly shape land-use and ecosystem services.

### *Option 3*: Bottom-up, diverse, multi-scale scenarios within a consistent global scenario context

IPBES could develop a diverse set of coordinated locally based scenarios that are linked to global scale scenarios. This proposal builds upon the existing work at the global level, but invests in developing new scenarios at the local scale, as well as in downscaling existing global scenarios. The rationale for this approach is that biodiversity and ecosystem services are strongly shaped by both local geography and local social-ecological dynamics (Reyers et al. 2013), which in turn are shaped by and reshape global drivers.

The impacts of global change vary across the world, and are shaped by local culture, preferences and wealth allocation. Local social and ecological diversity combine to produce responses that resonate out of their region. A top-down approach is likely to miss this heterogeneity and have difficulty engaging the diverse stakeholders, especially indigenous and local knowledge-holders.

A bottom-up approach can build on many local scenarios, stakeholder networks and local research capacities that are already in place, following examples such as the recent participatory biodiversity and conservation assessments by 60 communities in 20 different countries (Hall et al. 2015). IPBES could build upon these efforts and focus on the interactions among local trajectories and global dynamics. The regional assessments will highlight where it will be most useful to initiate locally based scenarios, given the current state of knowledge.

The advantage of this approach is that it would build upon existing analyses of large-scale global drivers, while acknowledging and using existing work to account for the social-ecological complexity of ecosystem services and biodiversity within landscapes. The challenge of such an approach would be coupling methods across scales, and managing the diversity of variables and knowledge systems needed to address different places. However, the experience of the MA suggests that it is easier to move from local to global than vice versa, because the knowledge and actions of local people, along with the characteristic behaviour of local ecosystems, have powerful effects on local dynamics and adaptation (Reid et al. 2006).

## Steps to developing an effective futures approach within IPBES

We believe that option 3 represents the best strategy for IPBES because it builds on existing global scenarios, while providing a defined space to develop new types of local and regional scenarios that address local heterogeneity, which is critical to ecosystem services and biodiversity. Furthermore, we believe this approach will provide most value to decision makers, other stakeholders and researchers engaged in environmental scenario development outside IPBES.

We propose five concrete steps that will utilise the strengths while overcoming potential weaknesses of option 3, as part of an effective, long-term scenario development process for IPBES in cooperation with the scientific community.

First, to ensure that the scenarios are relevant, credible, and useful it is essential for IPBES stakeholders, including indigenous communities and local peoples, to be involved in scenario definition and creation. To do this IPBES could establish a science-policy dialogue about scenarios to enable engagement and reflection with its stakeholders through its current assessments. The IPBES Stakeholder Engagement Forum, the International Indigenous Forum on Biodiversity and Ecosystem Services, and similar networks provide mechanisms ready to support engagement. This dialogue can be guided by on-going IPBES assessment activities, including the methodological assessment of scenarios and models of biodiversity and ecosystem services, as well as the on-going regional and thematic assessments.

Second, IPBES should learn from the experience of the MA scenario development process (Carpenter et al. 2006). The MA attempted to develop multi-scale scenarios, but largely failed due to local assessments beginning later, rather than the local scenarios feeding into the global. However, several sub-global assessments in the MA conduct more elaborate multi-scale scenarios in the Caribbean, Portugal and Southern Africa (Millennium Ecosystem Assessment 2005b). Such evaluation of MA experiences would clarify the specific opportunities and weaknesses a cross-scale scenario method poses for IPBES, as well as identify what key cross-scale biodiversity and ecosystem service issues the MA identified.

Third, IPBES could further explore how the framework of the SSPs, which addresses climate adaptation and mitigation challenges, can be extended to address biodiversity and ecosystem services issues relevant to IPBES. This effort would help clarify scenario options and needs for IPBES, and could also better connect the IPBES and IPCC research communities identifying knowledge gaps and opportunities for synthesis.

Fourth, IPBES should assess and identify effective participatory tools and processes that can bridge diverse knowledge systems in scenario processes. Such an assessment could build on IPBES on-going work with ILK, such as the multiple evidence base approach and existing reviews of local participatory scenario methods (Millennium Ecosystem Assessment 2005b; Oteros-Rozas et al. 2015). In particular, IPBES could play a role in bringing multiple knowledge systems together in scenario development and engage with science and policy communities, while also being useful for indigenous and other local knowledge holders.

Fifth, IPBES should consider how best to organize the scenario development process; this process is critical and challenging as its shapes what issues it can address. For global assessments there appears to be a trade-off between the need to anchor scenario development in a context that has the support and engagement of national governments and the requirement that scenarios address important, but sometimes politically sensitive issues that occur within or between nations. We recommend a scenario process similar to the MA and the SSPs; one that is organized with the support and endorsement of IPBES, but one that is also relatively independent from IPBES. Such a structure is also essential to enable effective engagement with politically contested indigenous and other local knowledge.

With the upcoming plenary, there is a window of opportunity for IPBES to embrace a long term scenario strategy that has the potential to significantly improve the relevance of future assessments on biodiversity and ecosystem services. We strongly urge IPBES to adopt a participatory, multi-scale scenario approach that captures the diversity of local social-ecological dynamics and builds understanding of interactions between global and local processes that intertwine to generate ecosystem services and human well-being.

## Footnotes

1 GDP, JA and RH are involved as authors in IPBES-assessments and RH is a member of the IPBES ILK Expert Taskforce. This article however, does not include any material associated with these roles and does not represent any official IPBES-position.
